# Correction: Effect of dose reduction of supplemental zinc for childhood diarrhea: study protocol for a double-masked, randomized, controlled trial in India and Tanzania

**DOI:** 10.1136/bmjpo-2019-000460corr1

**Published:** 2020-10-12

Somji SS, Dhingra P, Dhingra U, *et al*. Effect of dose reduction of supplemental zinc for childhood diarrhoea: study protocol for a double-masked, randomised controlled trial in India and Tanzania. *BMJ Paediatrics Open* 2019;3:e000460. doi: 10.1136/bmjpo-2019-000460

This article was previously published with the wrong licence. The correct licence for the paper is CC-BY.

