# Supplementary figures and images for: Effect of dose reduction of supplemental zinc for childhood diarrhoea: study protocol for a double-masked, randomised controlled trial in India and Tanzania
Source: BMJ Paediatr Open. 2019 Apr 24;3(1):e000460. doi: 10.1136/bmjpo-2019-000460 (PMC6542451; doi:10.1136/bmjpo-2019-000460)

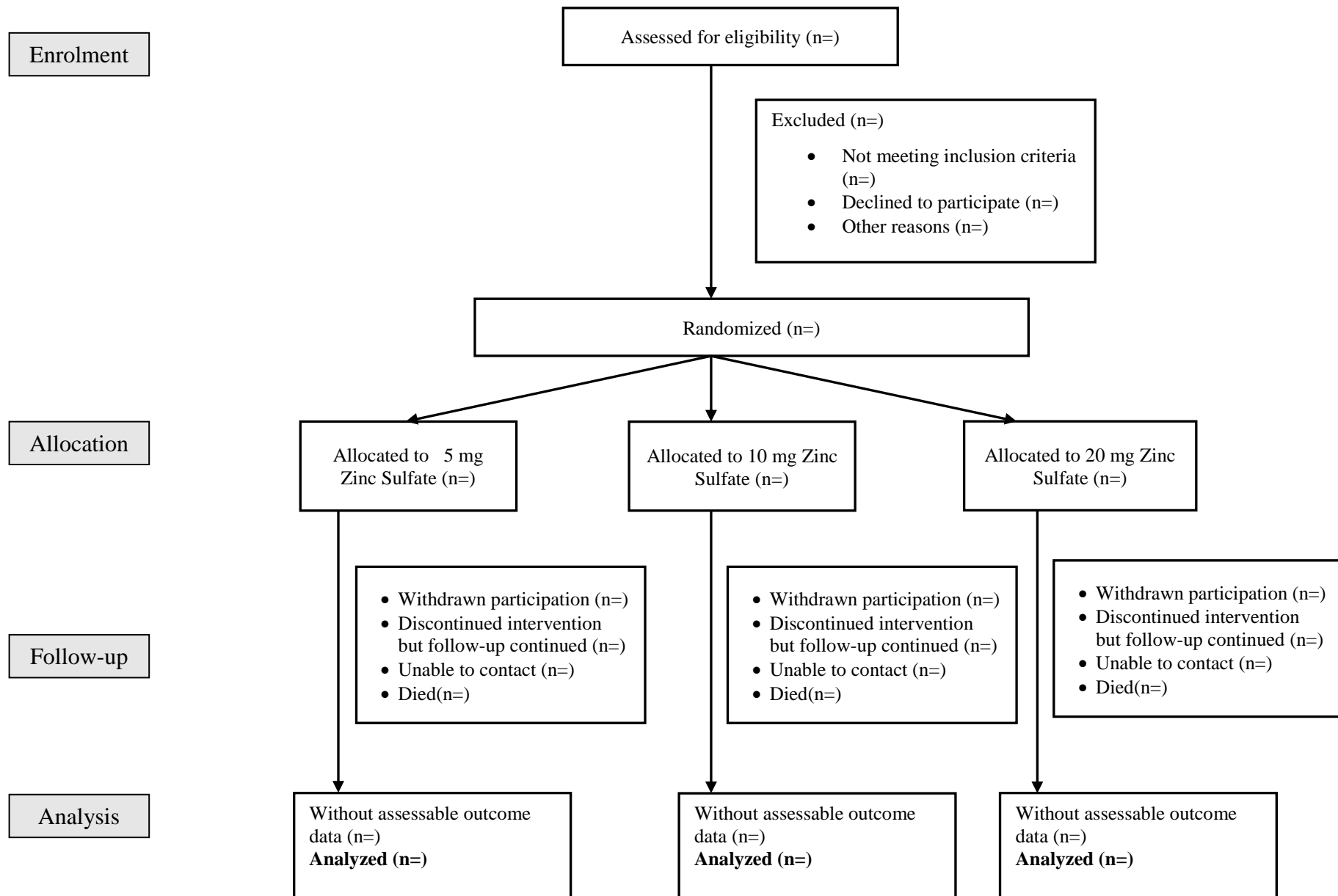

Figure 1: Consort Diagram

Supplement: Supplementary file 1 [file bmjpo-2019-000460supp001.pdf]
