# Supplementary material for: Effect of dose reduction of supplemental zinc for childhood diarrhoea: study protocol for a double-masked, randomised controlled trial in India and Tanzania
Source: BMJ Paediatr Open. 2019 Apr 24;3(1):e000460. doi: 10.1136/bmjpo-2019-000460 (PMC6542451; doi:10.1136/bmjpo-2019-000460)
Supplement: Supplementary file 2 [file bmjpo-2019-000460supp002.pdf]

	STUDY PERIOD										
	**Enrolment**	**Allocation**		**Post-allocation**							**Close-out**
**TIMEPOINT****	***0***	**0**	***Day 3***	***Day 5***	***Day 7***	***Day 10***	***Day 15***	***Day 21***	***Day 30***	***Day 45***	***Day 60***
**ENROLMENT:**											
**Eligibility screening**	X										
**Informed consent**	X										
Randomization	X										
**Allocation**		X									
**INTERVENTIONS:**											
Zinc 5 mg		X	—	—	—	—	—				
Zinc 10 mg		X	—	—	—	—	—				
Zinc 20 mg		X	—	—	—	—	—				
ORS/IV fluids		X									
**ASSESSMENTS:**											
Baseline demographics	X										
Infant anthropometrics	X										X
Breastfeeding status	X										
Rotavirus vaccination	X										
Antibiotic use for diarrhea treatment	X										
Zinc Supplement acceptability							X				
Infant morbidity									X	X	X
Blood plasma levels		X	X		X		X	X	X		
Duration of diarrhea			X	X	X	X	X				
Vomiting			X	X	X	X	X				

Fig. 2 Schedule of enrollment, interventions and assessments (SPIRIT Figure).
