# Supplementary material for: Effect of dose reduction of supplemental zinc for childhood diarrhoea: study protocol for a double-masked, randomised controlled trial in India and Tanzania
Source: BMJ Paediatr Open. 2019 Apr 24;3(1):e000460. doi: 10.1136/bmjpo-2019-000460 (PMC6542451; doi:10.1136/bmjpo-2019-000460)
Supplement: Supplementary file 4 [file bmjpo-2019-000460supp004.pdf]

TANZANIA FOOD AND DRUGS AUTHORITY

TFDA
Tanzania Food & Drugs Authority

0000034

CLINICAL TRIAL CERTIFICATE

(Made under section 61(2)(b)(ii) of the Tanzania Food, Drugs and Cosmetics Act, 2003)

Authorization No.TZ17CT002

This is to certify that, the clinical trial described below has been authorized to be conducted at **Temeke, Mwananyamala and Amana district hospitals Dar es Salaam** under the supervision of **Professor Karim P. Manji** as study Principal Investigator.

Study Title: **Establishing the optimal dose of therapeutic zinc supplementation for the treatment of acute diarrhea in under five children – a dose response trial in south Asian and a sub-Saharan African setting (aka ZTDT)**

Protocol Number: **ZTDT** Version Number: **3**

Protocol Date: **11th January 2017**

Study Sponsor: Rajiv Ball,
The World Health Organization,
Avenue Appia 20,
1211 Geneva 27,
SWITZERLAND

This certificate expires on: **12th February, 2020**

14/02/2017
....
Hiiti B. Sillo
DIRECTOR GENERAL

[signature]
....
Date

E-mail: info@tfda.go.tz
Tel: +255 22 2450512/24507551,
+255 65 844522/685701735,
+255 22 2452108/777700002
Website: www.tfda.go.tz
All letters should be addressed to the Director General
In reply please quote Our Ref No:

TFDA
Tanzania Food & Drugs Authority

Nelson Mandela Road
Mabibo External,
P.O. Box 77150,
DAR ES SALAAM,
TANZANIA.

Ref. No. TFDA0016/CTR/0015/03 **13th February, 2017**

Professor Karim P. Manji,
Principal Investigator,
Department of Paediatrics and Child Health,
Muhimbili University of Health and Allied Sciences,
P.O. Box 65001,
DAR ES SALAAM.

RE: APPROVAL TO CONDUCT A STUDY ENTITLED "ESTABLISHING THE OPTIMAL DOSE OF THERAPEUTIC ZINC SUPPLEMENTATION FOR THE TREATMENT OF ACUTE DIARRHEA IN UNDER FIVE CHILDREN – A DOSE RESPONSE TRIAL IN SOUTH ASIAN AND A SUB-SAHARAN AFRICAN SETTING (AKA ZTDT)"

1. Approval is hereby granted for you to conduct the above study.

2. The approved study sites are Temeke, Mwananyamala and Amana district hospitals-Dar es Salaam.

3. The approval is subject to the following conditions;

   a. Complying with all provisions of the Tanzania Food, Drugs and Cosmetics Act, Cap 219 and Tanzania Food, Drugs and Cosmetics (Clinical Trials Control) Regulations, 2013.

   b. Complying with the approved protocol ZTDT version 3 of 11th January 2017 and Informed consents swahili version of 22/12/2016 and English version of 26/09/2016.

   c. If for any reason the trial is prematurely terminated or suspended, a detailed written explanation must be submitted to TFDA not later than 15 days after the date of the discontinuance.

   d. The Authority may withdraw the approval already given if it is dissatisfied with the conduct of the study or there are breaches of any conditions prescribed in this letter.

   e. Six monthly progress and final reports should be submitted to TFDA, including interim analyses done by the Data Safety Monitoring Board (DSMB) or related Committee. The progress reports should be submitted within three weeks after the end of the period being reported and the final report within 60 days of conclusion of the trial.

MISSION

f. All relevant information, documents and records pertaining to the trial should be retained at the clinical trial site for a period of not less than 20 years after completion of a trial and made available upon request by TFDA.

g. Any amendment of the protocol, product or Investigators Brochure should be reported to TFDA and approval obtained before its implementation.

h. All serious adverse events should be reported in writing within 14 days and for fatal ones within 24 hours of their occurrence in any of the study sites.

i. Permits for Importation of Investigational Medicinal Product(s) should b\e obtained before importation. The product(s) should be inspected and approved at the port of entry.

j. Copies of publication(s) of any part of the study should be submitted.

4. The validity of this permit expires on **12th February, 2020.**

5. Looking forward to your continued cooperation.

Hhiti B. Sillo
DIRECTOR GENERAL

C.c: Rajiv Ball,
The World Health Organization ,
Avenue Appia 20,
1211 Geneva 27,
SWITZERLAND.

HBS/amf/am

CONDITIONS

1. Please ensure that the study is in compliance with the approved protocol, all provisions of the Tanzania Food, Drugs and Cosmetics Act, Cap 219 relating to clinical trials and the Tanzania Food, Drugs and Cosmetics (Clinical Trials Control) Regulations, 2013.

2. You should also ensure that;

   (i) All reports of analysis of safety data done by the Data Safety and Monitoring Board (DSMB) or equivalent committee and six monthly progress and final study reports are submitted to TFDA. Reports of DSMB should be submitted as soon as they are produced and within three weeks after the end of the period being reported and the final study report within 60 days of conclusion of the trial.

   (ii) You submit to TFDA copies of publications of any part of the study.

3. The Authority may withdraw the approval already given if it is dissatisfied with the conduct of the study or there are breaches of any condition prescribed in the law provision.

INSTITUTIONAL ETHICS COMMITTEE
SUBHARTI MEDICAL COLLEGE & HOSPITAL
SWAMI VIVEKANAND SUBHARTI UNIVERSITY

SUBHARTI PURAM, N.H. 58, DELHI–HARIDWAR, BY PASS ROAD, MEERUT. PIN-250005
Ph: 0121-2439056, 3058034 Ext. 2114 , Tele Fax: 0121-2439127 E-mail: subharatiinstitute@rediffmail.com www.subharti.org

No.SMC/ EC/2016/ 84

Date: 16.12.2016

CERTIFICATE

To

Dr. Sunil Sazawal
Executive Director Center for Public Health Kinetics
214 A, Basement Vinoha Puri Lajpat Nagar – II
New Delhi – 110024

Dear Dr. Sazawal

The Institutional Ethics Committee, Subharti Medical College & Hospital Meerut reviewed and discussed your research protocol entitled "**Establishing the optimal dose of therapeutic zinc supplementation for the treatment of acute diarrhea in under five children – a dose response trial in a South Asian and a Sub-Saharan African setting** " on 15.12.2016.

The following documents were reviewed:

1. Research Protocol version 3 & 4
2. Patient Information Sheet and Informed Consent Form in English & Hindi
3. Investigator's Brochure,
4. Proposed methods for patient accrual including advertisement (s) etc. proposed to be used for the purpose. N/A
5. Principal Investigator's current CV.
6. Insurance Policy / Compensation for participation and for serious adverse events occurring during the study participation. N/A
7. Investigator's Agreement with the Sponsor. N/A
8. Investigator's Undertaking (Appendix VII). N/A

The following members of the Ethics Committee were present at the meeting held on 15.12.2016

1.	Dr. Umesh Kumar Singh (Principal, KSCP)	Chairman
2.	Dr. P.P. Khosla (Professor & Head, Pharmacology)	Member
3.	Dr. Shashi Prateek (Professor Obs & Gyne.)	Member
4.	Dr. Sandeep Chaudhary (Professor Psychiatry)	Member
5.	Ms. Sarika Tyagi (Asst Professor, SPSIL)	Member
6.	Sh. V.K. Sharma (Bharat Vikash Parishad NGO)	Member
7.	Mr. Sardar Ahmed (Director Subharti Media Ltd.)	Member
8.	Dr Deshraj Singh (Ethicist& Philospher sanskriti Vibhag)	Member
9.	Dr A. K Srivastava (Professor & Head, Forensic Medicine)	Member Secretary

We approve the project to be conducted in its presented form. Ethics Committee also expects to be informed about the progress of the study, any SAE occurring in the course of the study, any changes in the protocol and patient information / informed consent and asks to be provided a copy of the final report.

Yours sincerely

(Dr. A. K. Srivastava)
Member Secretary
Ethics Committee.
