# Supplementary material for: Effect of dose reduction of supplemental zinc for childhood diarrhoea: study protocol for a double-masked, randomised controlled trial in India and Tanzania
Source: BMJ Paediatr Open. 2019 Apr 24;3(1):e000460. doi: 10.1136/bmjpo-2019-000460 (PMC6542451; doi:10.1136/bmjpo-2019-000460)
Supplement: Supplementary file 5 [file bmjpo-2019-000460supp005.pdf]

BILL & MELINDA
GATES *foundation*

PO Box 23350
Seattle, WA 98102, USA
V 206.709.3100
F 206.709.3180
www.gatesfoundation.org

October 23, 2015

64435 Zinc

Dr. Flavia Bustreo
Assistant Director-General, Family, Women's and Children's Health
World Health Organization
20 Avenue Appia
Geneva 27 CH-1211
Switzerland

Re: Global Health Grant OPP1135491
Project Name: Establishing the optimal dose of therapeutic zinc supplementation for the treatment of acute diarrhea in under five children - a dose response trial in a South Asian and a Sub-Saharan African setting.

Dear Dr. Bustreo:

The Bill & Melinda Gates Foundation (the "Foundation") is pleased to award the World Health Organization ("WHO") a project support grant in the amount of $2,865,011.00 for the period beginning on the date you sign this agreement (the "Start Date") to April 30, 2018 (the "Grant Period"). The purpose of the grant is to inform guidelines on therapeutic zinc supplementation by performing a dose-response evaluation of the impact of zinc in the care of children with diarrhea as described in your proposal dated October 20, 2015 and budget dated October 15, 2015.

Tax Status. You have confirmed that your organization is an international organization designated as such by Executive Order No. 10025 under the United States International Organizations Immunities Act (22 U.S.C. 288-288f).

Use of Grant Funds. The use of the grant funds and earnings thereon, is restricted solely to the purposes of the project described in your proposal and may not be expended, borrowed (inter-fund), pledged or transferred for reasons unassociated with the project. Grant funds may not be used for lobbying. You confirm that it is your organization's policy not to engage in, support or promote terrorism, either directly or indirectly through another organization or individual. The grant funds will be administered in accordance with the Financial Regulations and Rules, and financial and administrative rules and procedures of WHO. Any increases or decreases to a budget line for more than 10% must be approved by your assigned Program Officer in advance in writing. We understand that the funds will earn interest which will be affected to the purposes of the grant in accordance to WHO's policies and procedures. Grant funds will be used in accordance to WHO financial rules and regulations. Any portion of the grant funds unexpended or uncommitted at the end of the grant period, or at the time of termination of this agreement, and after all obligations by WHO prior to completion or termination have been fully liquidated, will revert to the Foundation.

Lobbying. You agree that no portion of the grant funds have been earmarked to support lobbying activities. As such, you agree that the budget for the Project does not include provisions for attempts to influence specific legislation either through (1) direct communications with any member or staff of a legislative body; or (2) urging members of the general public to contact members or staff of a legislative body. You further agree that the budget does not include provisions to influence the outcome of any specific public election.

Received on
MCA 199
24 JUIN 2016
ADG/FWC

Payment of Grant Funds. We will pay this grant via wire transfer to the following account:

A/C Name: World Health Organization
A/C No. 240-C0169920.3 (US$)
UBS AG
Case Postale 2600
CH-1211 Geneva 2
Switzerland
(IBAN Code: CH31 0024 0240 C016 9920 3)
(SWIFT Code: UBSWCHZH80A)

Payments and Reports. This table shows the deliverables (including reports) and milestones for this grant. Where indicated, the Foundation's payment is contingent on satisfaction of the listed deliverable and/or milestone. The Foundation may authorize changes to the payment and reporting schedules from time to time where appropriate. The Foundation will confirm any such changes in writing.

Payment Date	Payment Amount	Milestone or Deliverable	Due by	Reporting Period
Within 15 days after receipt of countersigned Agreement	$1,583,228.00	Countersigned Agreement	November 19, 2015	
		Satisfactory Progress Report	March 31, 2016	Start Date through December 31, 2015
May 2017	$1,281,783.00	Satisfactory Progress Report	March 31, 2017	January 1, 2016 through December 31, 2016
		Satisfactory Final Report	Within 90 days of End Date	Start Date to End Date
**AWARD TOTAL**	**$2,865,011.00**			

Reporting. We ask that you provide us with written narrative and financial reports in accordance with the schedule. All reports should clearly state the progress made in achieving the stated outcomes and deliverables. The Foundation will send you the report templates for this grant. Please use the reports to describe the progress you have made toward achieving the goals of the grant.

Record Maintenance and Inspection. All contributions to WHO are subject exclusively to its internal and external auditing procedures. The External Auditors' certification of accounts and audit reports is made available to the World Health Assembly on a biennial basis. WHO shall furnish to the Foundation, promptly upon their becoming available, a copy of WHO's Interim Financial Report issued at the end of the first year of a biennium, and its Financial Report and Audited Financial Statements, and the Report of the External Auditor to the World Health Assembly, issued at the end of a biennium and covering the full biennium. WHO agrees to visits from the Foundation's staff, if requested by the Foundation, to review technical progress of activities supported by this grant.

Compliance. If the Foundation is not satisfied with the progress of the project for which WHO has received grant funds, or the content of any written report, we will discuss in writing with WHO the reasons for the dissatisfaction. If thereafter no satisfactory agreement can be reached, the Foundation reserves the right to terminate this agreement.

Publication. You agree that you will prepare findings of the results of the work emerging from projects with Foundation grant funds for publication as soon as practical, consistent with the need to first secure intellectual property rights in a manner that benefit developing world interests.

Sub-Grants. Your proposal indicates a portion of the grant funds will be paid to sub-grantees to assist in the completion of the project. The Foundation makes no determination about the sub-grantees you choose and will not oversee their respective activities. Therefore, no implication should be made to investors, media or the general public that the Foundation supports the activities of any sub-grantee, and you agree to notify accordingly all sub-grantees you engage.

Clinical Trials. Since the Project will involve clinical trials on human subjects, a condition of this grant is your agreement that the appropriate Institution Review Boards ("IRBs") and ethical committees will review and approve the clinical protocols prior to trial initiation. You also agree to provide prompt notice to the Foundation if the facts and circumstances change regarding the approval status of the IRBs or ethical committees for any protocol(s).

Regulated Research Coverage. You confirm that all of the WHO's activities related to human subject research shall be guided by the Declaration of Helsinki (WMA 2000 and rev.2008 http://www.wma.net/e/policy/b3htm), the International Guidelines for Biomedical Research Involving Human Subjects (CIOMS, 2002), and the International Guidelines for Ethical Review of Epidemiological Studies (CIOMS 1991 being revised in 2008 http://www.cioms.ch/), as well as being subject to review and approval by the WHO Research Ethics Review Committee (ERC). You agree for each venue in which any part of the Project is conducted to require that sub-grantees or subcontractors comply with, assure and gain timely, appropriate prior approval for all activities subject to regulation and/or other types of required assurances or legal requirements, and that each venue will provide for appropriate data safety, monitoring and audit. As applicable, regulated activities and their documentation are to be conducted under the applicable international, national, and local standards. You further specifically agree that no funds will be expended to enroll human subjects until the necessary regulatory and ethical bodies' approvals are obtained. In keeping with general good clinical practice standards, subjects and the IRBs will be informed about what care and/or referrals will be available through participation in the study.

Grant Announcements; Public Reports and Use of Foundation Name and Logo. The Foundation will include information on this grant in our periodic public reports and may make grant information public at any time on its web page and as part of press releases, public reports, speeches, newsletters, and other public documents. If you wish to issue a press release or announcement regarding the award of this grant, you must obtain advance approval from the Foundation of the press release and the date of release. You also agree to obtain advance approval from the Foundation for any other use of the Foundation's name or logo. The Foundation requests an opportunity to review and comment on subsequent press releases or reports that are directly related to the grant. Please contact *Grantee.Comms@gatesfoundation.org* at least two weeks before any press release, announcement or other publication date.

Counterparts; Original. This Grant Agreement, including any amendments, may be executed in counterparts which, when taken together, will constitute one Grant Agreement. Copies of this Grant Agreement will be equally binding as originals and faxed or scanned and emailed counterpart signatures will be sufficient to evidence execution, though the Foundation may require you, the grantee, to deliver original signed documents.

Please sign and return this Grant Agreement to Grants Coordinator, Antjelina Newman, Antjelina.Newman@gatesfoundation.org. Please keep a copy for your records. If you have questions, please contact at David.Cook@gatesfoundation.org.

On behalf of the Foundation, may I extend every good wish for the success of your work.

Sincerely,

Anita K. M. Zaidi, MBBS, SM
Director, Enteric and Diarrheal Diseases

The World Health Organization, by its authorized representative agrees to the terms and conditions of this Grant Agreement.

Dr. Flavia Bustreo<br>Assistant Director-General<br>Family, Women's and Children's Health	27 October 2015<br>Date

On behalf of the Foundation, may I extend every good wish for the success of your work.

Sincerely,

Anita K. M. Zaidi, MBBS, SM
Director, Enteric and Diarrheal Diseases

The World Health Organization, by its authorized representative agrees to the terms and conditions of this Grant Agreement.

Dr. Flavia Bustreo
Assistant Director-General
Family, Women's and Children's Health

Date

Name	J. Simon	Coordinator	Director	MO	ADG
Unit	MRD	MRD	MCA	FWC	FWC
Date	26.10..15	26/10/15			
